# AI-enhanced non-invasive monitoring of critical biochemical analytes in perioperative care: a review of signal processing and clinical applications

**DOI:** 10.3389/fphys.2026.1794432

**Published:** 2026-04-16

**Authors:** Xiang Li, Jizhou Wang, Xiaoqin Jiang, Na Hu, Dongxu Chen

**Affiliations:** 1 Department of Anesthesiology, West China Second Hospital, Sichuan University, Chengdu, China; 2 Key Laboratory of Birth Defects and Related Diseases of Women and Children (Sichuan University), Ministry of Education, Chengdu, China; 3 School of Medical and Life Sciences, Chengdu University of Traditional Chinese Medicine, Chengdu, China; 4 Department of Anesthesiology, Chengdu Hi-Tech Zone Hospital for Women and Children, Chengdu, China; 5 Department of Anesthesiology, Sichuan Academy of Medical Sciences & Sichuan Provincial People's Hospital, University of Electronic Science and Technology of China, Chengdu, China

**Keywords:** critical biochemical analytes monitoring, internal environment, neural networks, non-invasive monitoring, perioperative care

## Abstract

Current perioperative monitoring of critical biochemical analytes predominantly relies on intermittent arterial blood gas analysis, which carries inherent risks of invasiveness and discontinuous data acquisition. Emerging evidence suggests that variations in electrocardiogram and photoplethysmogram waveforms may hold significant predictive value for detecting critical biochemical analytes changes. Advances in artificial intelligence analysis technologies have further accelerated the development of non-invasive monitoring tools, including real-time non-invasive blood glucose monitoring for diabetic patients and blood potassium monitoring for renal dialysis patients. This review highlights the urgent clinical need for non-invasive, continuous monitoring of the critical biochemical analytes during the perioperative period. It provides a comprehensive summary of current monitoring technologies and signals related to critical biochemical analytes, with a focus on their potential application in non-invasive blood gas monitoring. Based on existing evidence, key analytes such as serum potassium, serum calcium, lactate, and blood glucose have demonstrated robust research foundations and are the primary focus of this review. However, further clinical validation is urgently required to confirm their reliability and applicability in clinical settings. Integrating artificial intelligence with traditional monitoring systems has the potential to significantly enhance the precision, timeliness, and effectiveness of perioperative care. These findings suggest that AI-enhanced non-invasive monitoring could reduce unnecessary blood sampling while providing earlier detection of critical biochemical analytes disorder. Successful clinical translation requires standardized validation protocols and hardware-software co-development to address current limitations in measurement consistency and clinical workflow integration.

## Introduction

The real-time monitoring and regulation of changes in vital signs and the internal environment during the perioperative period are essential to ensuring patient safety. Globally, approximately 300 million surgical procedures are performed annually (Meara et al., 2015), highlighting the critical need for effective peri-aesthesia monitoring systems. Monitoring critical biochemical analytes primarily involves the assessment of electrolytes (e.g. potassium, calcium), metabolic parameters (e.g. glucose, lactate), and acid-base status (e.g. pH, PCO_2_). Disorders of the critical biochemical analytes are common, with over 50% of cases experiencing imbalances in blood potassium and calcium levels (Wang et al., 2018); 40% showing abnormalities in glucose and lactate levels, and more than 60% affected by acid-base disturbances (Dony et al., 2017). These disorders are closely associated with adverse patient outcomes. For example, hyperkalemia significantly increases early postoperative mortality in elderly patients undergoing orthopedic surgery (Norring-Agerskov et al., 2017). Similarly, perioperative hyperglycemia and hyperlactatemia are linked to a two- to three-fold higher risk of postoperative infections and kidney injury (He et al., 2023). Beyond short-term outcomes, these imbalances also impair long-term prognoses. For instance, metabolic acidosis during kidney transplantation has been linked to compromised renal function six months post-surgery (Tejchman et al., 2007). Consequently, effective peri-aesthesia monitoring and regulation of the critical biochemical analytes are crucial for improving patient safety and clinical outcomes.

Currently, perioperative monitoring currently relies heavily on intermittent and invasive blood gas analysis. This approach, which requires arterial blood samples and laboratory analysis, is often delayed by 20–60 minutes (Zi et al., 2023), even with in-room blood gas analyzers reducing this time to 3–15 minutes. Such delays can impair clinical decision-making and compromise patient safety. A study involving 860,000 surgical patients found that 10% of anesthesia-related deaths were related to inadequate monitoring, with 60% attributed to delays in detection and intervention (Arbous et al., 2001). Furthermore, invasive blood gas analysis carries inherent risks, particularly for pediatric patients, in whom arterial punctures are technically challenging and associated with high complication rates (Gleich et al., 2021).

Artificial intelligence (AI), driven by vast amounts of medical data, offers transformative potential in clinical analysis, diagnosis, and treatment. AI-powered medical devices, particularly those utilizing deep learning algorithms, have achieved significant advancements across various specialties, including cardiology, ophthalmology, respiratory medicine, and gastroenterology (Muehlematter et al., 2023). Despite these advancements, real-time, continuous, non-invasive perioperative monitoring of the critical biochemical analytes remains a significant unmet need. Addressing this challenge is pivotal for the evolution of perioperative monitoring and represents a cornerstone for advancing anesthesia management.

This review aims to explore recent advancements in non-invasive monitoring technologies for critical biochemical analytes, including glucose, lactate, and electrolytes, and evaluates the feasibility of non-invasive blood gas analysis in peri-anesthetic care. By addressing existing gaps, this review aimed to promote intelligent perioperative monitoring systems and enhance patient safety in surgical settings.

## Methods

This review included studies focusing on critical biochemical analytes, including but not limited to blood glucose, lactate, and electrolytes (e.g. serum potassium, serum calcium), etc. A systematic search of PubMed was performed on Feb 12, 2026 (**Supplementary 1**), with the search restricted to human studies. Letters, commentaries, editorials, and case reports were not included. The initial search yielded 645 titles and abstracts, which were independently reviewed by two reviewers. From these, 143 full-text articles were selected for detailed evaluation. Additionally, informal searches were conducted to address specific topics, and reference lists from previous reviews were examined to identify relevant studies. No formal criteria beyond those stated were applied for the inclusion of studies. The primary data extracted included study population, study design, type of signal source, analysis techniques, and predictive performance. Furthermore, we reviewed monitoring technologies and devices approved by the Food and Drug Administration (FDA) and the China National Medical Products Administration (NMPA) that are relevant to critical biochemical analytes monitoring. This review was conducted in adherence to the applicable SANRA (Scale for the Assessment of Narrative Review Articles) guidelines.

### Blood glucose monitoring

Hyperglycemia affects 80% of cardiac surgery patients, 20–40% of non-cardiac surgery patients, and 35% of patients with vascular disease (Long et al., 2019). Studies have shown that postoperative hyperglycemia significantly increases the risk of surgical site infections, reoperations, and mortality, regardless of diabetes status (Odds ratio [OR]: infection 2.0, reoperation 1.8, mortality 2.71) (Kwon et al., 2013). In extracorporeal circulation surgeries, glucose levels may need to be monitored at 10-minute intervals to facilitate early detection and timely intervention, improving outcomes (Kotagal et al., 2015). Finney’s study further underscored the importance of accurate and timely blood glucose monitoring by linking poor glucose control to increased mortality in critically ill patients (Finney, 2003).

Blood glucose monitoring methods can be categorized into invasive, minimally invasive, and non-invasive approaches based on the degree of tissue trauma involved. Invasive methods, such as capillary blood glucose testing via fingerstick, remain the most widely used in clinical practice. These methods are highly accurate but can cause discomfort and are impractical for frequent measurements. Minimally invasive techniques, such as glucose monitoring via interstitial fluid obtained through fine microneedles, reduce tissue damage compared to traditional invasive methods. However, these methods are susceptible to inaccuracies due to factors like sweat production and local environmental conditions (Kulcu et al., 2003). Driven by the growing demand for minimally invasive and portable monitoring devices, numerous advanced and well-established diagnostic technologies have been developed. **Table 1** presents FDA-approved minimally invasive glucose monitoring devices that are commercially available (Tierney et al., 2001; Barassi et al., 2015; Christiansen et al., 2017; Saha et al., 2017; Christiansen et al., 2018; Garg et al., 2022; Karinka et al., 2022). The earliest non-invasive glucose monitoring device, the GlucoWatch Biographer, was introduced in 2002. Resembling a conventional wristwatch, it paved the way for further developments in glucose monitoring technology. The Eversense Continuous Glucose Monitoring System (CGMS), for example, employs a minimally invasive procedure to implant a glucose sensor beneath the skin. The sensor predicts glucose concentration by detecting differences in fluorescence intensities, achieving an error rate of ≤20% in 93% of the data. The Guardian Connect device, approved by the FDA in 2018, achieves a 91.8% error rate of ≤20% in sensor-detected glucose data. The FreeStyle Libre series, updated to its third generation and approved by the FDA in May 2022, is marketed as the world’s thinnest and most accurate continuous glucose monitoring (CGM) device. The third-generation FreeStyle Libre is 70% smaller than its predecessor and achieves 93.2% of predictions within a 20% margin of error. The most recent advancement, the Dexcom G7 Continuous Glucose Monitoring System, received FDA approval in April 2024. The Dexcom G7 sensor, placed on the arm, demonstrates the highest accuracy among currently available devices, with 95.3% of monitoring data exhibiting an error rate of ≤20% and the lowest mean absolute relative difference (MARD) of 8.2% (A MARD <15% is considered indicative of accuracy comparable to fingerstick blood glucose testing.). Despite their high accuracy and continuous monitoring capabilities, these devices remain inherently invasive. Sensor patches require periodic replacement, and frequent recalibration with fingertip blood glucose tests is often necessary to ensure reliability.

**Table 1:** Minimally or non-invasive glucose monitoring device cleared for marketing by the FDA.

Product	Company	Year	Technology	Detection site	Performance	Participants	Sample sizes
Gluco Watch Biographer (Tierney et al., 2001)	Cygnus Technologies Inc.	2002	Reverse iontophoresis	Wrist	MD (-0.01 mmol/L) SD (2.31 mmol/L) MRD (3.7%) MARD (19%) Zones A+B (95.3%)	Diabetics	355
Freestyle libre (Karinka et al., 2022)	Abbott Diabetes Care Inc.	2016	Reverse iontophoresis	Upper arm	MARD (7.9%), RD (-3.1%)	Diabetics (>2 yrs), no skin lesions	332
Dexcom G7 (Garg et al., 2022)	Dexcom Inc.	2024	Reverse iontophoresis	Upper arm	MARD (8.2% for adults; 8.1% for children)	Diabetics (>2 yrs), no skin lesions	316
Smartest Glucowise (Saha et al., 2017)	Mediwise, Inc.	2013	mm-Wave Transmission spectroscopy	Hand	N/A	N/A	N/A
Eversense (Christiansen et al., 2018)	Senseonics Holdings, Inc.	2018	Fluorescence	Arm	zones A (92.8%) and B (6.5%).	Adults with diabetes	90
Guardian Connect (Christiansen et al., 2017)	Medtronic, Inc.	2018	Reverse iontophoresis	Abdomen	MARD (8.7%); SD (8.0%)	Diabetics between the ages of 14 and 75	118
OptiScanner® 5000 (Barassi et al., 2015)	Optiscan Biomedical Cor.	2020	Mid-infrared spectral technology	Arm	N/A	emergency patient	81

Fluorescence: An intermediary molecule (receptor) binds to glucose and emits fluorescence at a specific wavelength, thereby enabling the indirect monitoring of glucose levels; mm-Wave Transmission spectroscopy: A technology for indirectly monitoring blood glucose levels by detecting variations in the attenuation of microwaves’ signal propagation within tissues, which are caused by different blood glucose level concentrations; Mid-infrared spectral technology: A technology for indirectly monitoring blood glucose levels based on the principle that glucose-related molecules, such as glycated haemoglobin, cause variations in the optical properties of human tissues (e.g., fingertips) when exposed to light with wavelengths ranging from 2.5 to 10 micrometres; Zones A+B: The Clark Error Grid divides the error into five zones, each representing different clinical impacts: Zone A, this zone represents a very small deviation between the measured value and the true value, indicating highly accurate measurements that generally do not negatively affect treatment decisions; Zone B, The error in this zone is small, and although there may be some deviation, it has minimal impact on the patient’s treatment decisions; Reverse iontophoresis: A minimally invasive technique in which interstitial glucose is oxidised by enzymes (e.g. glucose oxidase Gox), and the microcurrents generated during the process react to the interstitial glucose concentration and, indirectly, to the blood glucose level concentration.

MARD, Mean Absolute Relative Difference, <10% is commonly considered high precision; MRD, Mean Residual Deviation, The average difference between the model’s predicted values and the actual values; MD, Mean Difference, The difference between the model’s predicted mean and the actual mean; N/A, Not Available; RD, Relative Difference, The difference between the predicted values and the actual values as a proportion of the actual values; SD, Standard Deviation, The dispersion of the model’s predicted values.

**Figure 1:**
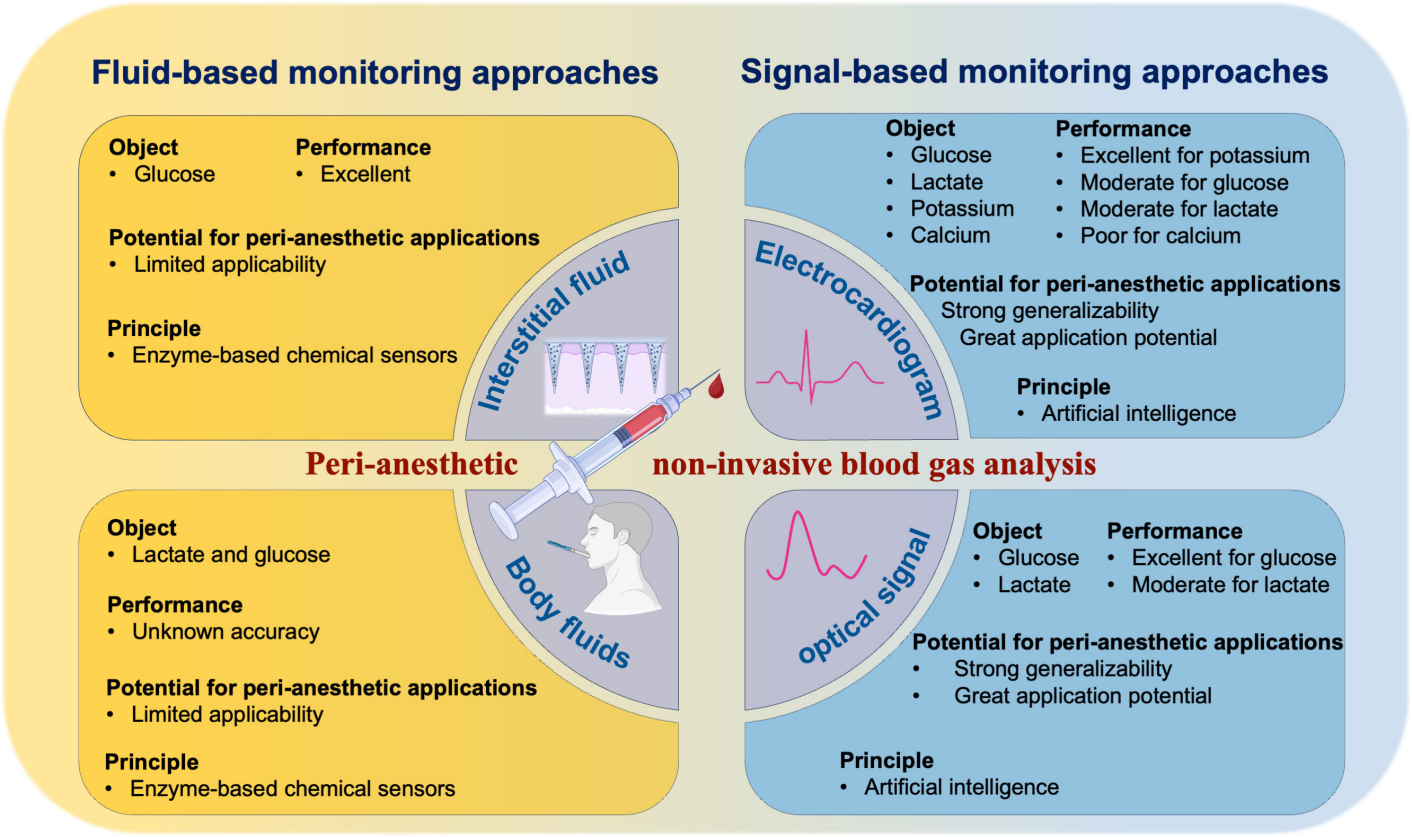
Comparison between different non-invasive monitoring methods for four targets.

Various non-invasive methods for blood glucose monitoring have been developed, including biosensor systems and technologies utilizing optical, microwave, and optical-acoustic combinations. Among these, the OptiScanner^®^ 5000, approved by the FDA in 2020, employs mid-infrared spectral technology to achieve non-invasive monitoring. Other devices include the C8 MediSensors device, which uses Raman spectroscopy, the Gwave device, which utilizes radio frequency technology, and the Glucontrol GC 300, which relies on near-infrared (NIR) technology. Recently, Zhang et al. developed a non-invasive blood glucose monitoring method based on improved Raman spectroscopy, demonstrating excellent monitoring performance (Zhang et al., 2025). While promising, the accuracy of these devices requires further validation in clinical settings. A novel direction in glucose monitoring leverages AI techniques to predict blood glucoses using physiological data such as electrocardiogram (ECG) and photoplethysmogram (PPG) signals (**Figure 1**). PPG signals contain rich information, with changes in blood glucose concentrations affecting tissue absorption of specific light wavelengths and influencing blood viscosity. Similarly, ECG waveforms have been shown to correlate with blood glucose, with parameters such as heart rate (HR), heart rate variability (HRV), and segments like ST and QT demonstrating a 92% sensitivity for glucose prediction (Tobore et al., 2019) Recent advancements in AI, particularly the application of neural networks such as Convolutional Neural Networks (CNN) and Deep Neural Networks (DNN), have significantly enhanced the accuracy of non-invasive glucose monitoring. For instance, a study involving 21 participants collected 103 days of ECG and PPG signals. The proposed model achieved excellent monitoring performance with a root-mean-square error (RMSE) of 1.49 mmol/L, a mean absolute relative difference (MARD) of 13.42%, and Zone A+B coverage of 99.49% in tenfold cross-validation (Li et al., 2024). **Table 2** provides an overview of recent studies exploring non-invasive blood glucose monitoring methods (Krishnan et al., 2020; Kownacka et al., 2018; Habbu et al., 2019; Joshi et al., 2020; Porumb et al., 2020; Tsai et al., 2020; Zhang et al., 2020; Deng et al., 2021; Lee and Lee, 2021; Li et al., 2021; Sempionatto et al., 2021; Nie et al., 2023; Li et al., 2024; Saha et al., 2024; Pors et al., 2025; Zhang et al., 2025; Zhang et al., 2026).

**Table 2:** Non-invasive blood glucose monitoring technology.

Author	Signal Source	Algorithm	Clarke error grid analysis (%)		Accuracy (%)	Sensitivity (%)	Specificity (%)	MARD (%)	Validation Method
			zone A	zone B					
(Habbu et al., 2019)	PPG	NN	85.20	13.60	N/A	N/A	N/A	N/A	N/A
(Joshi et al., 2020)	NIR	DNN and MPR	94.00	6.00	N/A	N/A	N/A	6.07	External
(Krishnan et al., 2020)	PPG	ML	N/A	N/A	94.23	N/A	N/A	N/A	N/A
(Tsai et al., 2020)	PPG	ML	N/A	N/A	79.5	N/A	N/A	N/A	Internal
(Zhang et al., 2020)	PPG	ML	N/A	N/A	81.49	N/A	N/A	N/A	N/A
(Porumb et al., 2020)	ECG	CNN	N/A	N/A	89.3 ± 4.5	94.5 ± 7.7	87.2 ± 5.5	N/A	Internal
Internal validation		CNN + RNN	N/A	N/A	90.0 ± 5.2	87.4 ± 10.4	92.2 ± 3.8	N/A
(Lee and Lee, 2021)	PPG	DNN	88.57	8	84.29	N/A	N/A	N/A	External
(Deng et al., 2021)	PPG	DNN	N/A	N/A	90.25	N/A	N/A	N/A	External
(Li et al., 2021)	ECG	CNN	N/A	N/A	88	N/A	N/A	N/A	N/A
(Nie et al., 2023)	PPG	ML	89.60	10.40	N/A	N/A	N/A	N/A	Internal
(Li et al., 2024)	ECG and PPG	Multimodal Framework*	80.90	19.40	N/A	N/A	N/A	13.42	Internal
(Sempionatto et al., 2021)	Sweat	enzymatic biosensor	81.2	18.8	N/A	N/A	N/A	7.79	Internal
(Saha et al., 2024)	Sweat	enzymatic biosensor	84.7(fingertip) 79.35(forearm)	15.3(fingertip) 20.65(forearm)	N/A	N/A	N/A	10.56 (fingertip) 13.17 (forearm)	Internal
(Kownacka et al., 2018)	Tears	electrochemical sensor	70	25	N/A	N/A	N/A	12.5	N/A
(Zhang et al., 2025)	PPG	MPR	81.4	18	N/A	N/A	N/A	14.3	External
(Pors et al., 2025)	PPG	CNN	90	10	N/A	N/A	N/A	8.9	N/A
(Zhang et al., 2026)	Sweat	enzymatic biosensor	N/A	N/A	N/A	N/A	N/A	N/A	External

*Multimodal framework: the multimodal framework performs three-level fusion. First, ECG and PPG signals are collected and coupled, Second, the temporal statistical features and spatial morphological features in the ECG and PPG signals are extracted and determined with three feature selection techniques and compressed by deep neural networks (DNNs) Lastly, weight-based Choquet integral multimodal fusion is integrated.

Acc, accuracy; CNN, Convolutional neural network; DNN, Deep neural networks; ISF, interstitial fluid; MARD, Mean Absolute Relative Difference; MPR, Multiple Polynomial Regression; ML, machine learning; NIR, Near Infrared; N/A, not available/given; PPG, Photoplethysmography; Sen, sensitivity; Spe, speciality.

Tissue fluid glucose levels, such as those measured in interstitial fluid (ISF), often lag behind blood glucose. Consequently, most device manufacturers note that ISF glucose monitoring may not accurately reflect rapid changes in blood glucose. Beyond physiological signals like ECG and PPG, bodily fluids such as saliva, sweat, and tears are being explored as alternative mediums for glucose monitoring. However, the correlation between glucose levels in these fluids and blood glucose remains inconsistent (Patel et al., 2015). For example, experimental devices have been developed for saliva glucose estimation, such as one affixed to dried teeth (Tseng et al., 2018), and for tear glucose monitoring using contact lenses (Farandos et al., 2015). Saha developed a sweat-based glucose monitoring device demonstrating promising accuracy (MARD ~10.56%), but further clinical trials are needed to validate its efficacy (Saha et al., 2024). Unfortunately, these technologies are still in the experimental stages, primarily limited to animal studies, and significant challenges persist in ensuring sensor safety and continuous usability.

Despite advancements, non-invasive glucose monitoring methods have yet to find practical application in the operating room. Current FDA-approved minimally invasive glucose monitoring devices meet the requirements for continuous and dynamic perioperative monitoring. However, pre-market clinical trials for these devices have primarily focused on diabetic patients, often excluding those with severe comorbidities or visible skin lesions. Additional limitations include prolonged sensor calibration times (up to several hours) and high costs, which may hinder widespread adoption. ISF-based methods, while minimally invasive and broadly applicable, suffer from reduced accuracy and are highly susceptible to environmental factors. For instance, anesthesia medications that suppress glandular secretion and constant low temperatures in operating room can significantly interfere with measurements, leading to poor precision (Singh and Jha, 2019). Diagnostic research addressing these limitations remains scarce (Kulcu et al., 2003). Blood glucose monitoring methods leveraging AI offer significant potential. Assuming sufficient monitoring accuracy, AI-assisted approaches provide timeliness, continuous and dynamic measurement, non-invasiveness, and ease of deployment. Physiological signals such as ECG and PPG, combined with neural network models, can enable real-time and dynamic blood glucose prediction. These methods impose minimal additional burden on clinical workflows and patients while offering continuous monitoring capabilities. It is worth paying attention to in the future.

### Lactate monitoring

For patients without evidence of tissue hypoperfusion, lactate monitoring can help identify other underlying factors, providing a more accurate assessment of the clinical condition. Changes in lactate clearance rates have been identified as independent predictors of mortality risk (De Wever et al., 2004). Consequently, continuous and dynamic lactate monitoring is essential. Existing non-invasive methods can be broadly categorized into two types: Fluid-based approaches and Signal-based approaches (**Table 3**) (Petropoulos et al., 2016; Lin et al., 2018; Mason et al., 2018; Huang et al., 2019; Budidha et al., 2020; Olaetxea et al., 2020; Yuzer et al., 2022; Huang et al., 2023; Rabost-Garcia et al., 2023; Zi et al., 2023).

**Table 3:** Non-invasive lactate monitoring technology.

Author	Signal source	Method	Performance	Participants	Validation method	Purpose
Yuzer (Yuzer et al., 2022)	Sweat	Colorimetric + CNN	Accuracy: 0.99 Precision: 0.99 Recall: 0.99 F1-score: 0.99 ROC-AUC: 0.99	General population	N/A	Monitoring
Lin (Lin et al., 2018)	Tear	Electrochemical	N/A	N/A	N/A	Monitoring
Petropoulos (Petropoulos et al., 2016)	Saliva	Electrochemical	N/A	Athletes	N/A	Monitoring
Olaetxea (Olaetxea et al., 2020)	Blood	Raman Spectroscopy	RMSEP: 1.25 mM *R*^2^: 0.96	N/A	N/A	Monitoring
Budidha (Budidha et al., 2020)	NIRS	Partial Least-Squares regression analysis and leave-one-out cross-validation	*R*^2^: 0.976 RMSECV: 0.89mmol/L	N/A	N/A	Monitoring
Mason (Mason et al., 2018)	Blood	Electromagnetic + NN	*R*^2^: 0.78	General population	N/A	Monitoring
Zeng (Zi et al., 2023)	ECG	CNN	Accuracy: 80.27% Sensitivity: 79.93% AUC: 0.92 F1-score: 0.8	Patient	External	Monitoring
Huang (Huang et al., 2019)	Anthropometric elements	ML	SD (min): 0.1	Healthy subjects	Internal	Monitoring
Huang (Huang et al., 2023)	ECG	CNN+ANN	N/A	Healthy subjects	External	Monitoring
Rabost (Rabost-Garcia et al., 2023)	Sweat	ML	RMSE: 1.56mM	Athletes	External	Diagnostic equivalence

CNN, Convolutional neural network; ML, Machine learning; N/A, not available/given; R², A measure of how well the data fit a regression model. It shows the percentage of variance explained by the model, ranging from 0 (no fit) to 1 (perfect fit); RMSE, Root Mean Square Error; RMSECV, Root Mean Square Error of Cross-Validation; RMSEP, Root Mean Square Error of Prediction; F1-score: A metric that combines precision and recall into one number, balancing false positives and false negatives. It ranges from 0 to 1, with 1 being the best.

Fluid-based monitoring approaches: Portable sweat sensors are capable of analyzing metabolites such as glucose, lactate, and electrolytes (Gao et al., 2016). Electrochemical sensors are commonly used to predict lactate levels in sweat. For example, Ruggeri et al. combined colorimetric methods with machine learning techniques to develop a predictive model with excellent performance (Ruggeri et al., 2023). Additionally, sensors have been designed to measure lactate levels in tears (Lin et al., 2018) and saliva (Petropoulos et al., 2016); although these methods lack sufficient diagnostic experimental data to support their clinical use.

Signal-based monitoring approaches: Near-infrared (NIR) spectroscopy has been investigated for its potential to measure lactate concentrations by evaluating light absorption. For instance, Karthik demonstrated a strong correlation between lactate levels and light absorption in sodium lactate solutions, simulating whole blood (Budidha et al., 2020). Similarly, Huang proposed that lactate concentrations might correlate with cardiopulmonary variables and exercise intensity, developing a machine-learning model with excellent fitting performance under low to moderate intensities (Yeragani et al., 1996). Studies also suggest that lactate levels impact myocardial electrophysiological activity, with Zeng employing deep neural networks to analyze twelve-lead ECG data, achieving an accuracy of 80.27% in predicting lactate concentrations (Zi et al., 2023).

Despite these advancements, there is a notable lack of research on non-invasive lactate monitoring in perioperative settings (**Figure 1**). Fluid-based methods, such as those relying on sweat, are primarily used in sports medicine. The correlation between blood lactate levels and lactate levels in bodily fluids remains inconsistent, with some studies suggesting a relationship (Karpova et al., 2020) while others do not (Klous et al., 2021). A few studies have directly focused on blood lactate concentration as the primary monitoring target, with Rabost directly investigating blood lactate levels, meeting the monitoring requirements for the peri-anesthetic period (Rabost-Garcia et al., 2023). However. factors such as operating room temperature, cholinergic receptor antagonist use, and variability in critical biochemical analytes further limit the utility of fluid-based methods in perioperative care. Signal-based techniques, such as NIR spectroscopy, face challenges related to interference from substances like hemoglobin, which affect absorbance. These limitations highlight the need for innovative approaches. Non-invasive lactate monitoring using neural networks based on ECG data holds promise. Current models show good predictive performance in laboratory settings, impose no additional burden on patients, and are easily integrated into routine ECG monitoring. As neural network algorithms and computational power improve, the accuracy and clinical applicability of these methods are expected to advance.

### Electrolytes: potassium

Hyperkalemia is strongly associated with poor clinical outcomes and, in severe cases, can lead to arrhythmias, heart failure, or death. A 2019 study in Japan reported a 6.8% incidence of hyperkalemia in the general population (Kashihara et al., 2019), while studies in China estimate a prevalence of 2–3%, with a misdiagnosis rate as high as 89% (Lai et al., 2021). Despite its clinical significance, the frequency of serum potassium monitoring during the perioperative period remains critically insufficient.

Conventional potassium monitoring relies on invasive blood sampling, which is limited by its inability to provide continuous, dynamic assessments. A portable, continuous, minimally invasive potassium monitoring device (CKM™) has been developed, but robust clinical validation is still lacking. Initial research has explored non-invasive potassium monitoring using PPG for classification (Miller et al., 2023), though most techniques rely on ECG. For example, potassium concentration is mainly related to the amplitude of the T wave, while calcium concentration correlates with the QT interval. This relationship was recognized early and utilized for empirical estimation of potassium levels in patients. However, empirical predictions are highly inaccurate. Approximately 81% of hyperkalemic patients’ ECGs are not correctly interpreted by clinicians (Rafique et al., 2020). One study revealed that the proportion of correctly identified hyperkalemic ECGs was only 34%-43%. Additionally, some normokalemic individuals exhibit ECG features resembling hyperkalemia, and hyperkalemic patients may present atypical ECG patterns, further complicating diagnosis. To avoid these limitations, mathematical models incorporating ECG features—such as T wave height, width, and slope—were developed (Attia et al., 2016), achieving sensitivity and specificity above 80%. However, these models often overemphasize local ECG features, introducing selection bias and reducing predictive accuracy at extreme potassium concentrations (Corsi et al., 2017). Enhancing model parameters, such as applying periodic component analysis, has been shown to improve prediction accuracy, although this significantly increases the computational workload associated with traditional mathematical data analysis methods.

Neural network algorithms offer a promising alternative to address these challenges (**Figure 1**). These models can process vast datasets and automatically identify critical ECG features for potassium prediction, mitigating issues of selection bias. Bachmann’s study demonstrated the superiority of DNN models over traditional computational models, highlighting their ability to optimize predictions across varying electrolyte concentrations (von Bachmann et al., 2024).

**Table 4** provides an overview of techniques for monitoring potassium and calcium disorders, including non-invasive methods for serum potassium assessment (Corsi et al., 2012; Severi et al., 2009; Corsi et al., 2012; Attia et al., 2016; Corsi et al., 2017; Velagapudi et al., 2017; Lin et al., 2020; Regolisti et al., 2020; Kwon et al., 2021; Wang et al., 2021; Kim et al., 2023; Miller et al., 2023; Harmon et al., 2024; von Bachmann et al., 2024; Shang et al., 2025). Early approaches relied on simple mathematical models with limited accuracy. Velagapudi’s study achieved a sensitivity of only 63% using a basic mathematical model (Velagapudi et al., 2017). In contrast, Corsi and colleagues developed models for quantitative analysis of serum potassium levels, achieving a mean absolute deviation of approximately 0.5 mmol/L (Corsi et al., 2012). Compared to these methods, potassium monitoring using neural network-assisted techniques has significantly improved predictive performance. For instance, in Harmon’s study, the AI-ECG method demonstrated sensitivity and specificity exceeding 80%, with a negative predictive value surpassing 99%, highlighting its utility as a preliminary screening tool for electrolyte imbalances (Harmon et al., 2024). Similarly, Attia’s study used single-lead ECG for potassium prediction, achieving a prediction error within 10% of actual values (Attia et al., 2016). These advancements position AI-ECG as a promising technology for clinical applications. While ECG-based potassium level monitoring has already shown potential in managing dialysis patients, its adaptation for perioperative care has yet to be confirmed through dedicated research. Neural network-assisted potassium monitoring using ECG data offers several advantages: 1) Non-invasive and continuous monitoring: Eliminates the need for invasive blood sampling while providing dynamic, real-time assessments; 2) Convenience and cost-effectiveness: ECG monitoring is widely available and requires minimal additional resources. 3) Broad applicability: The flexibility of this approach allows for innovative applications, such as potassium prediction using ECG images captured via smartphones (Kim et al., 2023). Further research is needed to validate these techniques in perioperative settings and explore their full potential in broader clinical applications.

**Table 4:** Non-invasive methods for monitoring blood potassium levels.

a: Non-invasive methods for monitoring blood potassium and calcium levels. (Classification models)											
Author	Algorithm	Signal source	Classification tasks	Sensitivity, (%)	Specificity, (%)	AUC	N_Pat_, n	ECGs, n		Validation method	Purpose
Miller (Miller et al., 2023)	ML	PPG	Hyperkalemia	53	N/A	0.877	N/A	N/A		N/A	Screening
			Hypokalemia	0.93	N/A	0.877	N/A	N/A			
Von (von Bachmann et al., 2024)	DNN	ECG	Hyperkalemia	N/A	N/A	0.892	290889	N/A		Internal	Monitoring
			Hypokalaemia	N/A	N/A	0.809		N/A			
Velagapudi (Velagapudi et al., 2017)	SMM	ECG	Hyperkalemia	63	84	0.78	107	236		N/A	Monitoring
Regolisti (Regolisti et al., 2020)	LOOCV	T-wave	Hyperkalemia and hypokalaemia	N/A	N/A	0.74	149	N/A		External	Screening
Kim (Kim et al., 2023)	AI	ECG	Hyperkalemia	79.7	93.4	0.898-0.902	125	N/A		N/A	Diagnostic equivalence
Harmon (Harmon et al., 2024)	AI	ECG	Hyperkalemia	80	80	0.88	40128	N/A		Internal	Screening
Lin (Lin et al., 2020)	CNN	ECG	Hyperkalemia	83.3	97.8	0.958	N/A	40180		External	Screening
			Hypokalaemia	96.7	93.3	0.926	N/A	N/A			
Wang (Wang et al., 2021)	CNN	ECG	Hypokalaemia	71.4	77.1	0.80	N/A	8630		External	Screening
Kwon (Kwon et al., 2021)	CNN	ECG	Hyperkalemia	90.1	85	0.945	48365	83449		External	Monitoring
			Hypokalaemia	89.3	70.4	0.866					
Shang (Shang et al., 2025)	CNN	ECG	Hyperkalemia	98.3	97.88	N/A	500	N/A		Internal	Screening
*Von (von Bachmann et al., 2024)	DNN	ECG	Hypercalcaemia	N/A	N/A	0.66	125970	N/A		Internal	Monitoring
			Hypocalcaemia	N/A	N/A	0.779					
*Kwon (Kwon et al., 2021)	CNN	ECG	Hypercalcaemia	90.9	52.1	0.905	N/A	N/A		External	Monitoring
			Hypocalcaemia	89.1	84.7	0.901					

b: Non-invasive methods for monitoring blood potassium levels. (Quantitative models)											
Author	Model	Method	Classification tasks	Mean error	Mean absolute error		SD		Validation method		Purpose
Corsi (Corsi et al., 2017)	SMM	ECG	Qualitative	-0.09 ± 0.59 mM	0.46 ± 0.39 mM		N/A		Internal		Monitoring
Severi (Severi et al., 2009)	SMM	ECG	Qualitative	-0.03 mM	0.49 ± 0.16 mM		0.83 mM		N/A		Monitoring
Corsi (Corsi et al., 2012)	SMM	ECG	Qualitative	0.05 mM	0.43 ± 0.28 mM		0.5 mM		Internal		Monitoring
Corsi (Corsi et al., 2012)	SMM	ECG	Qualitative	-0.09 ± 0.59 mM	0.48 ± 0.35 mM		N/A		Internal		Monitoring
Attia (Attia et al., 2016)	SMM	ECG	Qualitative	0.41 mM	0.5 ± 0.42 mM		N/A		External		Monitoring

*Test targets calcium disorders. CNN, Convolutional neural network; DNN, Deep neural networks; DLM, Deep learning models; N_Pat_, number of patients; N/A, not available/given; LOOCV, linear and logistic regression and leave-one-out cross-validation; ROC, A performance metric for classification models. ROC (Receiver Operating Characteristic) curve plots true positive rate vs. false positive rate, and AUC (Area Under the Curve) measures the overall ability of the model to discriminate between classes. AUC ranges from 0 to 1, with 1 being perfect. SD, standard deviation; SMM, simple mathematical model.

### Electrolytes: calcium

Calcium ions play a vital role in the body, regulating cellular processes through kinase cascade reactions, enzyme activation, and other signaling pathways. Mild or gradual changes in calcium levels are often asymptomatic, with significant gastrointestinal or neuropsychiatric symptoms manifesting only when imbalances are severe or rapid. Both hypercalcemia and hypocalcemia are associated with poor clinical outcomes. Studies indicate that hypocalcemia increases mortality rates threefold in patients with pulmonary embolism (Murthi et al., 2021). Additionally, early calcium monitoring and intervention in trauma patients can significantly reduce transfusion requirements and the incidence of trauma-induced coagulopathy (De Robertis et al., 2015).

Research on non-invasive calcium monitoring remains limited (**Figure 1**). Recent advancements in ECG analysis, supported by deep learning technologies, have provided a new avenue for non-invasive monitoring. Calcium ion concentration affects the ECG by altering action potential duration and the QT interval. However, the performance of deep learning models for predicting blood calcium levels has been inconsistent (**Table 4**). For example, in Kwon’s study, the specificity for hypercalcemia prediction was only 52% (Kim et al., 2023). This limitation arises due to the highly concentrated normal range of calcium ions in healthy individuals. During model training, minimal fluctuations in calcium ion levels result in negligible ECG changes, leading to suboptimal fitting performance (Corsi et al., 2012). Additionally, the complex physiological mechanisms involved in calcium regulation may further limit the accuracy of these models.

While current ECG-based deep learning models for blood calcium prediction may currently be better suited as initial screening tools rather than definitive diagnostic instruments, they hold potential for specific applications. Continuous and dynamic monitoring of calcium levels could be particularly valuable in improving outcomes for high-risk populations, such as patients with severe trauma, hyperparathyroidism, or endocrine tumors. Future research should focus on optimizing model parameters, integrating multimodal data sources, such as by employing hybrid models combining ECG and Fluid-based signals and conducting robust clinical validation to enhance the utility of non-invasive calcium monitoring. These efforts could pave the way for more reliable and accessible tools for early detection and management of calcium imbalances.

### Limitation

This review assessed several emerging technologies for non-invasive blood gas analysis and identified deep learning–assisted monitoring based on ECG or PPG signals as the most promising direction. However, given the rapid evolution of deep learning technologies, the findings and discussions presented here may soon require updating. A notable limitation in current non-invasive monitoring research is the inconsistent reporting of performance metrics. Although Clarke and Parkes Error Grid Analyses and MARD are the accepted standards for assessing clinical accuracy, reliance on binary metrics (e.g., sensitivity and specificity) persists in some studies. Such metrics are prone to boundary effects and may increase the susceptibility to reporting bias. Currently, a consensus on specific performance metrics for intraoperative monitoring technologies is lacking. Nevertheless, drawing from recent FDA “Breakthrough Device Designations”, we observe that an AUC around 0.9 and a MARD <10% serve as de facto references (U.S. Food and Drug Administration, 2018a; U.S. Food and Drug Administration, 2018b; U.S. Food and Drug Administration, 2021; U.S. Food and Drug Administration, 2022; U.S. Food and Drug Administration, 2024). In high-stakes environments like the perioperative period, sensitivity demands are typically even higher (>90%). The formulation of dedicated standards for these scenarios remains a crucial objective for future studies. Furthermore, some studies included in this review suffer from small sample sizes, making them highly susceptible to overfitting—a challenge particularly pronounced in models utilizing deep learning technologies (Miotto et al., 2018). Because deep learning networks process physiological signals with complex temporal characteristics, calculating a traditional ‘minimum sample size’ as done in conventional clinical research is often impractical. Therefore, solutions must be implemented at the algorithmic level. For models with limited training data, transfer learning can be employed to mitigate the risk of overfitting. Additionally, data augmentation techniques—such as adding noise, introducing baseline wander to the original signals, or utilizing Generative Adversarial Networks (GANs)—can be strategically used to expand the training set (Iwana and Uchida, 2021). During the model training phase, strategies like dropout and early stopping are also highly effective in preventing overfitting and enhancing model generalization. Importantly, due to their inherent ‘black-box’ nature, current deep learning models for non-invasive monitoring often lack clinical interpretability. To facilitate the clinical translation of these technologies and earn the trust of physicians, future studies must prioritize Explainable AI (XAI) techniques (Amann et al., 2020). For instance, Class Activation Mapping (e.g., Grad-CAM and Saliency Maps) can be utilized to generate heatmaps, verifying whether the model’s predictive focus aligns with established medical knowledge—such as explicitly highlighting peaked T-waves on an ECG when predicting hyperkalemia (Kwon et al., 2021). Additionally, SHapley Additive exPlanations (SHAP) can be implemented to quantify the specific impact and contribution of various input parameters on the final prediction, thereby making the decision-making process transparent. In addition, this review primarily focused on metabolic indicators (e.g., glucose and lactate) and electrolytes (e.g., potassium and calcium) based on currently available evidence. Other key internal environmental parameters—such as pH, bicarbonate, and base excess—remain insufficiently studied due to limited data. Future research should prioritize these underrepresented parameters, starting with those that are already well characterized and progressively expanding to a broader spectrum of internal environment components. Most of the studies included in this review evaluated non-invasive monitoring performance within specific populations and under clinical conditions.

Existing research on non-invasive monitoring is predominantly segmented: glucose for daily diabetes management, lactate for athletic performance, and potassium for routine clinical care. Few studies target the perioperative phase, where unique confounders exist. Anesthesia, surgical stress, and blood loss can significantly impact cardiac electrophysiology and peripheral perfusion, challenging the signal integrity of ECG and PPG. Similarly, sweat-based lactate sensing is vulnerable to hypothermia and hemodynamic instability. It remains to be proven whether the performance of these technologies in non-surgical settings can be successfully extrapolated to the complex perioperative context. In future clinical practice, it will be essential to account for regional and ethnic differences, variations in baseline health status, differences in data-acquisition devices, and the influence of complex surgical procedures on monitoring accuracy and reliability. By addressing these gaps, the development of comprehensive, real-time, non-invasive blood gas monitoring systems can significantly enhance patient care and clinical outcomes.

## Conclusion

This review highlights four critical biochemical parameters—glucose, lactate, potassium, and calcium—by examining current non-invasive monitoring alternatives, comparing the advantages and limitations of various methods, and evaluating their suitability for the perioperative environment. While technologies such as micro-needling, ISF monitoring, and Raman spectroscopy have demonstrated rapid advancements, non-invasive monitoring of critical biochemical analytes using physiological signals from the body surface, such as ECG and PPG, shows significant promise for perioperative applications due to its unique advantages in this context. Future efforts should aim to expand the range of monitored parameters by extracting additional information from ECG and PPG signals, maximizing the capabilities of neural networks in processing large datasets, and adopting multimodal monitoring approaches. This could enable cross-validation and supplementation of key parameters, including those not directly measurable (e.g., pH, sodium levels), to create a more comprehensive monitoring system. However, this approach is not without limitations. High-quality data are essential for effective model training, and significant variability exists in the predictive performance of different models. Challenges also include the complexity of interpreting results, the potential for overfitting, and the need for robust validation in diverse clinical settings. Furthermore, among all FDA-approved AI medical devices, the majority focus on image analysis, highlighting the relative underdevelopment of AI applications in continuous physiological monitoring. Despite these challenges, advancements in peri-anesthetic digital intelligence hold immense potential to transform the field. Continuous, real-time non-invasive monitoring of internal environments could greatly enhance patient safety, minimize the risks associated with invasive procedures, and elevate the standard of perioperative care. By addressing the current limitations and fostering innovation in AI-driven technologies, the future of perioperative monitoring is poised to become more accurate, efficient, and patient-centered.

## Supplementary material

Supplementary 1 Search strategies for non-invasive monitoring methods for four targets.
