# Supplementary material for: AI-enhanced non-invasive monitoring of critical biochemical analytes in perioperative care: a review of signal processing and clinical applications
Source: Front Physiol. 2026 Apr 16;17:1794432. doi: 10.3389/fphys.2026.1794432 (PMC13128380; doi:10.3389/fphys.2026.1794432)
Supplement: Supplementary 1 — Search strategies for non-invasive monitoring methods for four targets. [file DataSheet1.docx]

**Search strategies**

**Search date:** February 2026

**PUBMED**

**Total: 1666**

1 Glucose[Title/Abstract]

2 Glycemi*[Title/Abstract]

3 #1 or #2

4 Electrolyte[Title/Abstract]

5 (Calcium[Title/Abstract]) OR (Magnesium[Title/Abstract])

6 #4 or #5

7 (Lactate[Title/Abstract]) AND (Lactic[Title/Abstract])

8 #3 or #6 or #7

9 ((Blood[Title/Abstract]) OR (Plasma[Title/Abstract])) OR (Serum[Title/Abstract])

10 #8 and #9

11 Blood gas[Title/Abstract]

12 #10 or #11

13 (Non-invasive[Title/Abstract]) OR (noninvasive[Title/Abstract])

14 Monitor*[Title/Abstract]

15 #12 and #13 and #14
